# Peroxisomes are platforms for cytomegalovirus’ evasion from the cellular immune response

**DOI:** 10.1038/srep26028

**Published:** 2016-05-16

**Authors:** Ana Cristina Magalhães, Ana Rita Ferreira, Sílvia Gomes, Marta Vieira, Ana Gouveia, Isabel Valença, Markus Islinger, Rute Nascimento, Michael Schrader, Jonathan C. Kagan, Daniela Ribeiro

**Affiliations:** 1 Institute for Research in Biomedicine – iBiMED, Department of Medical Sciences & Department of Biology, University of Aveiro, Aveiro, Portugal; 2 Neuroanatomy, Center for Biomedicine and Medical Technology Mannheim, University of Heidelberg, Heidelberg, Germany; 3 Infections and Immunity Laboratory, Instituto Gulbenkian de Ciência, Oeiras, Portugal; 4 College of Life and Environmental Sciences, Biosciences, University of Exeter, Exeter, Devon, UK; 5 Division of Gastroenterology, Boston Children’s Hospital and Harvard Medical School, Boston, MA, USA

## Abstract

The human cytomegalovirus developed distinct evasion mechanisms from the cellular antiviral response involving vMIA, a virally-encoded protein that is not only able to prevent cellular apoptosis but also to inhibit signalling downstream from mitochondrial MAVS. vMIA has been shown to localize at mitochondria and to trigger their fragmentation, a phenomenon proven to be essential for the signalling inhibition. Here, we demonstrate that vMIA is also localized at peroxisomes, induces their fragmentation and inhibits the peroxisomal-dependent antiviral signalling pathway. Importantly, we demonstrate that peroxisomal fragmentation is not essential for vMIA to specifically inhibit signalling downstream the peroxisomal MAVS. We also show that vMIA interacts with the cytoplasmic chaperone Pex19, suggesting that the virus has developed a strategy to highjack the peroxisomal membrane proteins’ transport machinery. Furthermore, we show that vMIA is able to specifically interact with the peroxisomal MAVS. Our results demonstrate that peroxisomes constitute a platform for evasion of the cellular antiviral response and that the human cytomegalovirus has developed a mechanism by which it is able to specifically evade the peroxisomal MAVS-dependent antiviral signalling.

The human cytomegalovirus (HCMV) is a large enveloped virus with double-stranded DNA genome that belongs to the *Herpesviridae* family. HCMV is a highly widespread pathogen that has been described as one of the major causes of birth defects, when acute infection occurs during pregnancy, and opportunistic diseases in immunocompromised patients^1^. HCMV has the ability to establish a state of latency and persist indefinitely in the host despite the continuously induced antiviral immune responses^2^.

Apoptosis is one of the first lines of defence against viral infections. With a slow replication cycle, HCMV depends on the sustained cell viability^2^ and, in order to prevent the premature death of infected cells, the virus has evolved various strategies to block apoptotic signalling pathways and subvert the host antiviral response^3^,^4^. HCMV encodes vMIA (mitochondria-localized inhibitor of apoptosis, also named pUL37 × 1) that plays an important role on the inhibition of apoptosis^5^,^6^. vMIA prevents the formation of the mitochondrial permeability transition pore, the release of cytochrome c and pro-apoptotic factors into the cytoplasm as well as the activation of executioner caspases^4^. Although the mechanism involved is still somewhat controversial, it was shown that vMIA interferes with Bax and triggers the blockage of the mitochondrial outer membrane permeabilization^6^,^7^. Among other functions, vMIA also induces calcium (Ca^2+^) efflux from the endoplasmic reticulum (ER), regulates viral early gene expression and disrupts F-actin^8^.

vMIA has also been shown to inhibit the cellular antiviral response by dampening signalling downstream from the mitochondrial MAVS (mitochondrial antiviral signalling adaptor) and triggering mitochondria fragmentation, a phenomenon proven to be essential for this signalling inhibition^9^,^10^.

MAVS-dependent antiviral signalling is activated by the recognition of the viral genome by the soluble RNA helicases RIG-I-like receptors (RLR) such as the retinoic acid inducible gene-I (RIG-I) and the melanoma differentiation-associated gene-5 (MDA-5). Upon viral stimulation, these proteins undergo a conformational change, leading to their dimerization and interaction with MAVS through their CARD domains^11^. This leads to a signalling cascade that culminates with the induction of type-I interferons (IFN) and IFN-stimulated genes (ISGs) that may function as direct antiviral effectors, preventing important steps in viral propagation. It has been suggested that vMIA’s inhibition of the MAVS-dependent signalling may be due to a reduction of the interaction between MAVS and the cytoplasmic DNA sensor STING (stimulator of interferon genes), an ER protein that was reported to be associated with MAVS and to be important for type-I IFN production after viral infection^12^,^13^. It has been suggested that, by inducing mitochondrial fragmentation, vMIA affects the association between this organelle and the ER, disturbs the MAVS-STING interaction and, consequently, dampens type-I IFN signalling and ISGs production^9^,^14^.

Dixit *et al*.^15^ have demonstrated that MAVS is also localized at peroxisomes and that peroxisomal and mitochondrial MAVS assume complementing functions within the antiviral response. The peroxisomal MAVS induces the rapid expression of ISGs, conferring short-term protection, while the mitochondrial MAVS activates an interferon-dependent signalling pathway with delayed kinetics that amplifies and stabilizes the antiviral response^15^. Peroxisomes represent a class of ubiquitous and essential single-membrane bound subcellular organelles that fulfil important metabolic functions in, among others, lipid and reactive oxygen species (ROS) metabolism^16^,^17^. Like mitochondria, peroxisomes are dynamic and their protein composition, morphology and abundance is tightly regulated upon external stimuli to maintain cellular homeostasis^18^,^19^. The discovery of the presence of MAVS at peroxisomes has added a novel function to this organelle in cellular antiviral signalling, expanding their impact on health and disease.

In this manuscript we investigated the possibility that HCMV would have developed a mechanism through which it could specifically interfere with the peroxisomal MAVS-dependent signalling pathway. Our results indeed demonstrate that vMIA is also localized at peroxisomes and dampens the peroxisomal MAVS-dependent production of ISGs. Furthermore, we demonstrate that vMIA interacts with the peroxisomal MAVS and induces peroxisomal fragmentation, a morphological change that, unlike for mitochondria, does not seem to be relevant for the antiviral signalling inhibition.

## Results

### Cytomegalovirus’ protein vMIA localizes at peroxisomes and induces their fragmentation

**Figure 1:**
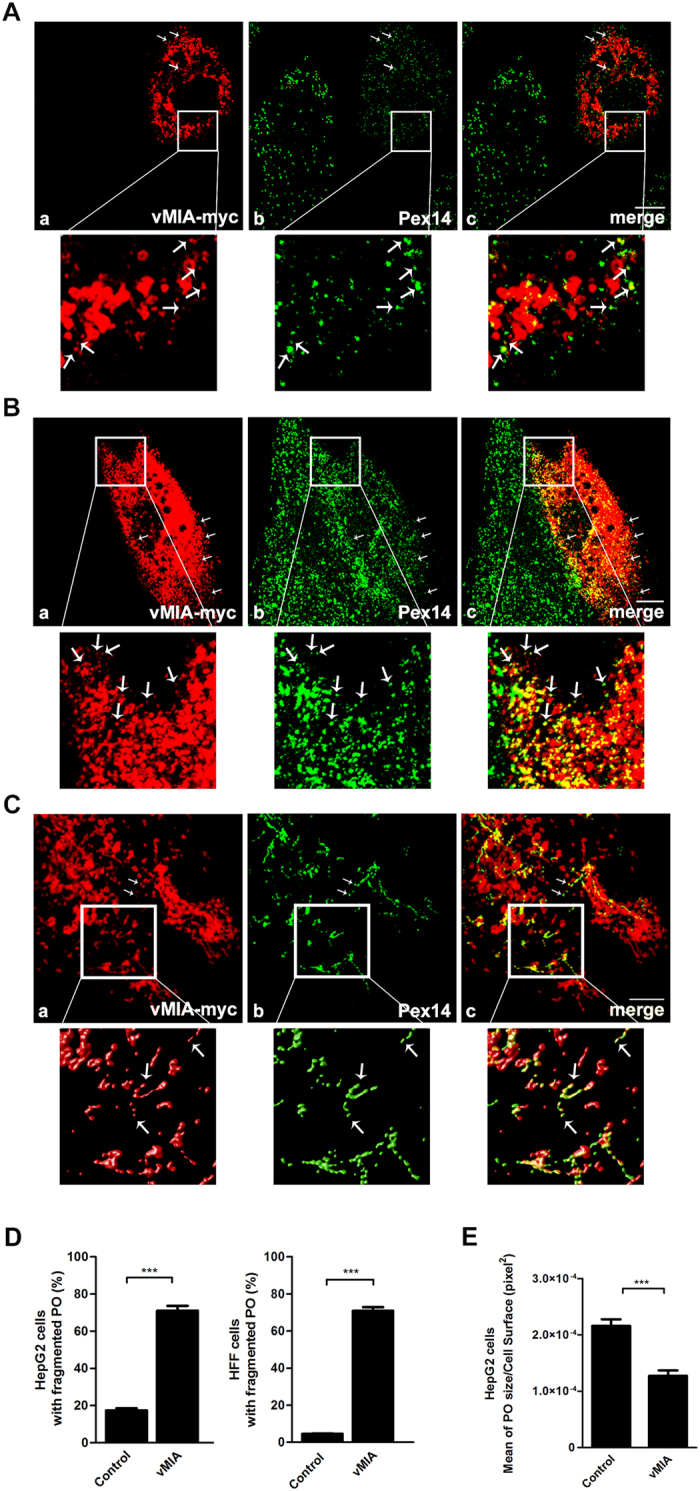
vMIA localizes at peroxisomes and causes their fragmentation. **(A**) (a-c) vMIA intracellular localization in HepG2 cells (a) vMIA-myc, (b) Pex14 and (c) merge image of a and b. (**B**) (a-c) vMIA intracellular localization in HFF cells (a) vMIA-myc, (b) Pex14 and (c) merge image of a and b. (**C**) (a-c) vMIA intracellular localization in DLP1-patient cells. (a) vMIA-myc, (b) Pex14 and (c) merge image of a and b. The images presented in the zoom insets from panel (**C**) are the result of deconvolution and 3D rendering analysis. Confocal images from immunofluorescence staining. Bars represent 10 μm. Arrows represent co-localization loci. (**D**) Quantification analysis of peroxisome morphology in the presence and absence of vMIA in HepG2 and HFF cells. We considered cells containing “fragmented peroxisomes” as those whose peroxisomes were significantly smaller and in higher number when compared to the control cells. Data represents the means ± SEM of three independent experiments. Error bars represent SEM. ***p < 0.001 in a Student’s t test. (**E**) Quantification analysis of peroxisomal area (pixel^2^) in the presence or absence of vMIA in HepG2 cells, using the Spot Detector plug-in from Icy Bioimage Analysis Software. Data represents the means ± SEM of three independent experiments. Error bars represent SEM. ***p < 0.001 in a Student’s t test.

The RLR adaptor protein MAVS is localized at mitochondria and peroxisomes (as well as at the mitochondrial-associated endoplasmic reticulum membranes, MAM), and these organelles act in concert to establish the cellular antiviral response to viral infections^15^,^20^,^21^. vMIA (Fig. S1A) has been shown to induce mitochondrial fragmentation^6^,^10^ and modulate the mitochondrial MAVS-dependent signalling^9^. As peroxisomes and mitochondria share many of their membrane proteins, including the main components of their division machinery, we wondered whether vMIA would also localize at peroxisomes and interfere with the antiviral signalling pathway that is established at this organelle. To that end, HepG2 cells (human hepatocyte cell model) as well as HFF cells (human foreskin fibroblasts, a specific cell type that is commonly infected by HCMV) were transfected with myc-tagged vMIA and, after 24 h, subjected to immunolocalization analysis with antibodies against myc and the peroxisomal marker Pex14. In addition to displaying the expected localization pattern at fragmented mitochondria (Fig. 1A,B and Fig. S1D), we found vMIA to be also localized at peroxisomes, both in HepG2 (Fig. 1A) and HFF cells (Fig. 1B). An analysis of the Manders’ co-localization coefficients indicates that 6.69% of the vMIA co-localizes with the peroxisomal marker in HepG2 cells and 26.12% in HFF cells.

In order to more clearly demonstrate the presence of vMIA at peroxisomes we performed similar transfection and immunolocalization analysis in fibroblasts that present bigger and hypertubulated peroxisomes. These cells (which we here name DLP1-patient cells) were isolated from a patient with an heterozygous, dominant-negative mutation in the DLP1 gene^22^ and present a dramatic defect on peroxisomal and mitochondrial fission, exhibiting mainly hypertubulated organelles. As shown in Fig. 1C (where the zoom insets present the results obtained with deconvolution and 3D rendering analyses), vMIA clearly localizes at the hypertubulated peroxisomes.

Interestingly, as shown in Fig. 1A,B where one can compare a transfected and a non-transfected cell for each of the cell lines, vMIA overexpression induced a significant peroxisomal fragmentation: peroxisomes appear smaller and in higher number. In order to support this observation, we performed statistical analysis where six hundred cells (of each of the cell lines) from three independent experiments were analysed for each condition, taking into account the size/shape and number of their peroxisomes. We considered cells containing “fragmented peroxisomes” as those whose peroxisomes were significantly smaller and in higher number when compared to the control cells (differences in fluorescence intensities were taken into account for each cell and experiment). As shown in Fig. 1D, 71% of the HepG2 cells expressing vMIA contained fragmented peroxisomes, while only 17% of control cells displayed this phenotype. Similarly, the results for HFF cells show an increase on the number of cells with fragmented peroxisomes from about 4% (in control cells) to 70% upon vMIA overexpression (Fig. 1D). Using the Spot Detector plug-in from Icy Bioimage Analysis Software^23^, we confirmed that, upon vMIA overexpression in HepG2 cells, there was a decrease in the mean surface area of each peroxisome (Fig. 1E). In all the cells exhibiting a peroxisomal fragmentation, a fragmentation of the mitochondrial network was also observed (Fig. S1D).

In all these experiments there was no difference on Pex14 expression levels (Fig. S1B and C).

**Figure 2:**
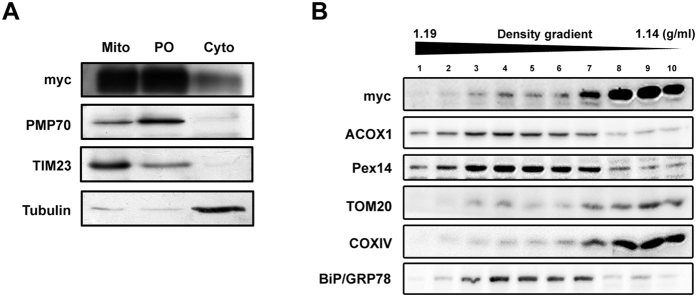
Biochemical analysis of vMIA intracellular localization. (**A**) Western blot analysis of the presence of vMIA in cytosolic (Cyto), mitochondrial (Mito) and peroxisomal-enriched (PO) fractions, upon differential centrifugation of HFF lysates. PMP70, TIM23 and tubulin were used as peroxisomal, mitochondrial and cytosolic markers, respectively. (**B**) Western blot analysis of the localization of vMIA after density gradient centrifugation of HepG2 lysates. ACOX1 and Pex14 are used as peroxisomal markers, TOM20 and COXIV are used as mitochondrial markers and BiP/GRP78 as ER marker.

In order to perform a biochemical analysis that would complement the results obtained with the immunofluorescence analyses and, as the current methodologies do not allow the preparation of pure peroxisomal fractions from cell cultures, we have performed differential centrifugation experiments with lysates from HFF cells transfected with vMIA-myc and obtained a fraction that (although presenting some degree of contamination with light mitochondria and small vesicles such as lysosomes and endosomes) is highly enriched in peroxisomes (Fig. 2A, PO). We found that vMIA is present at the enriched peroxisomal fractions (Fig. 2A), confirming and complementing the results obtained with the immunofluorescence analyses. We have also performed a density gradient centrifugation (Fig. 2B) with HepG2 cells and obtained similar results: the majority of vMIA co-migrates with the mitochondrial markers but is also present at the fraction where the peroxisomal markers are concentrated (Fig. 2B, lane 4). As expected, and similarly to the fractionation experiment, there is some degree of contamination of this fraction with mitochondria. In fact, besides being a common drawback on the analysis of peroxisomal fractions with the currently available methodologies, this is enhanced by the presence of vMIA: as this protein induces mitochondrial fragmentation, there will be an increase on the presence of small mitochondria at the peroxisomal fractions. The density gradient results show also some level of co-migration of the peroxisomal and ER-markers. This is, however, irrelevant for the vMIA localization analysis as we, and others, have never observed the presence of this protein at this organelle.

**Figure 3:**
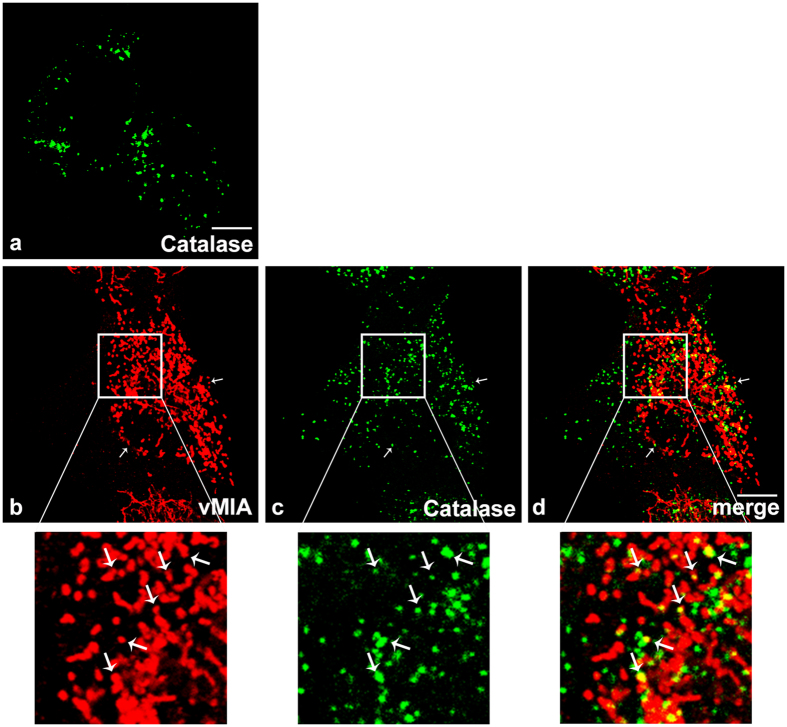
vMIA localization upon HCMV infection. (**a**) Representative image of peroxisomal morphology in uninfected HFF cells, stained with catalase. (**b–d**) HFF cells infected with HCMV, 8 h post-infection. (**b**) vMIA, (**c**) catalase and (**d**) merge image of b and c. Arrows indicate co-localization loci. Bar represents 10 μm.

In order to determine whether virally-produced vMIA is also present at peroxisomes, we analysed the localization of this protein upon infection of HFF cells with HCMV. HFF cells where infected with the HCMV AD169 strain and, 8 h after infection, were subjected to immunofluorescence analysis with antibodies against vMIA and the peroxisomal marker catalase. As shown in Fig. 3, the HCMV-produced vMIA is not only localized at fragmented mitochondria but is also present at peroxisomes. We have once again calculated the Manders’ co-localization coefficient and the results show that 11.65% of vMIA co-localizes with the peroxisomal marker. Interestingly, the infected cells (Fig. 3b–d) show some level of peroxisomal fragmentation (defined by a higher number of smaller peroxisomes) when compared to non-infected cells (Fig. 3a), similarly to what was observed upon vMIA overexpression.

Overall, these results demonstrate that vMIA localizes at peroxisomes and regulates peroxisome morphology.

### vMIA travels to peroxisomes via interaction with Pex19

**Figure 4:**
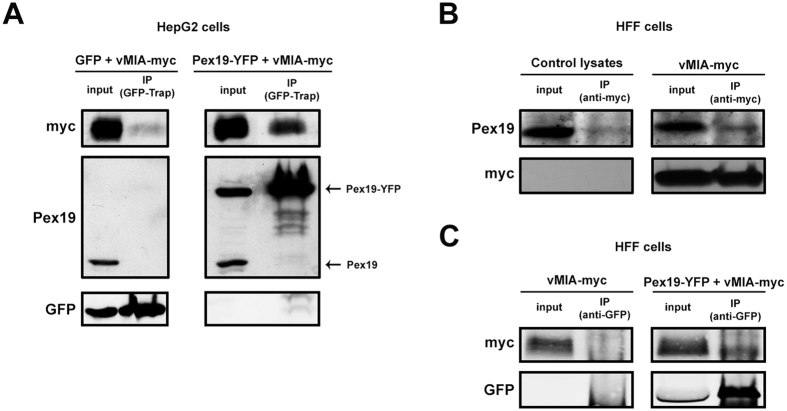
vMIA interacts with Pex19. (**A**) Co-immunoprecipitation analysis of the interaction between overexpressed Pex19-YFP and vMIA-myc in HepG2 cells. The pull-down was performed using GFP-Trap M kit. Negative control was performed by immunoprecipitating cells overexpressing GFP and vMIA-myc. Western blot was performed with antibodies against myc, Pex19 and GFP. Input represents total cell lysate and IP represents the immunoprecipitation. Arrows indicate endogenous Pex19 and the transfected Pex19-YFP. (**B**) Co-immunoprecipitation analysis of the interaction between overexpressed vMIA-myc and endogenous Pex19 in HFF cells. The pull-down was performed using an antibody against myc. Negative control was performed by immunoprecipitating non-transfected cells. Western blot was performed with antibodies against myc and Pex19. Input represents total cell lysate and IP represents the immunoprecipitation. (**C**) Co-immunoprecipitation analysis of the interaction between overexpressed Pex19-YFP and vMIA-myc in HFF cells. The pull-down was performed using an antibody against GFP. Negative control was performed by immunoprecipitating cells overexpressing vMIA-myc. Western blot was performed with antibodies against myc and GFP. Input represents total cell lysate and IP represents the immunoprecipitation.

The novel localization of vMIA at peroxisomes raises the question of how this viral protein is actually delivered to this organelle. Peroxisomal membrane proteins are mostly transported by interaction with the Pex19 cytosolic chaperone, which directs them to the organelle’s membrane by interacting with Pex3^24^,^25^,^26^. To test the hypothesis that HCMV could highjack the peroxisomal transport machinery in order to localize vMIA at this organelle, we analysed a possible interaction between this protein and Pex19. To that end, we co-transfected vMIA-myc and Pex19-YFP (for 24 h) in HepG2 cells and performed co-immunoprecipitation analyses. As shown in Fig. 4A, vMIA interacts with Pex19 (with a 7-fold increase when comparing the bands from the immunoprecipitation -IP- and the control). Similar analyses were performed in HFF cells where vMIA-myc was overexpressed (for 24 h) and its co-immunoprecipitation with the endogenous Pex19 was analysed. Fig. 4B shows that, also in HFF, vMIA interacts with Pex19 (although with a lower 3-fold increase when comparing the IP and control bands). The interactions of peroxisomal membrane proteins with Pex19 are very transient and sometimes quite difficult to show, depending on the cell type. Hence, to complement and solidify these results, we have also demonstrated this interaction in HFF cells by co-immunoprecipitation upon overexpression of vMIA-myc and Pex19-YFP (for 24 h) (Fig. 4C), obtaining a 5-fold increase when comparing the IP and control bands. Altogether these results support a model whereby Pex19 binds and chaperones this viral protein to the peroxisomal membranes.

### vMIA interacts with the peroxisomal MAVS and inhibits the peroxisomal-dependent antiviral signalling pathway

**Figure 5:**
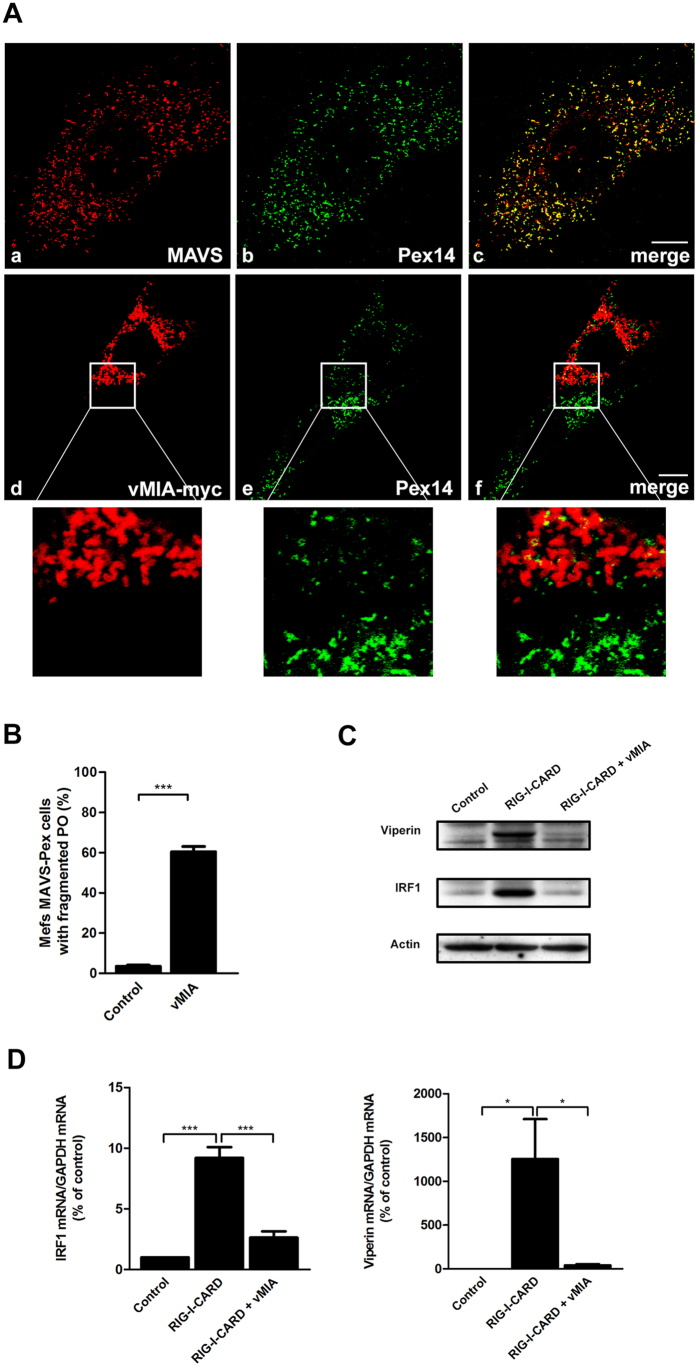
vMIA inhibits the peroxisomal-dependent antiviral signalling. (**A**) (a–c) MAVS intracellular localization in Mefs MAVS-Pex cells (a) MAVS, (b) Pex14 and (c) merge image of a and b. (d–f) localization of transfected vMIA in Mefs MAVS-Pex cells (d) vMIA-myc, (e) Pex14 and (f) merge image of d and e. Confocal images from immunofluorescence staining. Bar represents 10 μm. (**B**) Quantification analysis of peroxisome morphology in the presence and absence of vMIA in Mefs MAVS-Pex cells. Data represents the means ± SEM of three independent experiments. Error bars represent SEM. ***p < 0.001 in a Student’s t test. (**C**) Western blot analysis of the production of IRF1 and viperin in Mefs MAVS-Pex cells stimulated with GFP-RIG-I-CARD in the presence or absence of vMIA. Representative image of three independent experiments. Actin was used as a loading control. (**D**) RT-qPCR analysis of IRF1 and viperin mRNA in Mefs MAVS-Pex cells stimulated with GFP-RIG-I-CARD in the presence or absence of vMIA. GAPDH was used as control. Data represents the means ± SEM of three independent experiments. Error bars represent SEM. *p < 0.05 and ***p < 0.001 in one-way ANOVA, with Bonferroni’s post test.

vMIA has been suggested to impede signalling downstream from MAVS^9^. However, in these studies, neither the peroxisomal MAVS nor vMIA localization at peroxisomes were taken into account. To determine whether vMIA has any significant effect on the signalling downstream from the peroxisomal MAVS, we have expressed vMIA-myc in Mefs cells that contain MAVS solely at peroxisomes (Mefs MAVS-Pex cells, Fig. 5A)^15^. In order to find out whether these cells would respond to vMIA overexpression in a similar way as HepG2 and HFF we analysed peroxisome fragmentation by immunolocalization. It is important to note that the peroxisomes in these cells are in general more elongated than in HepG2 or HFF cells. Fig. 5A d-f shows examples of peroxisome morphology in un-transfected and vMIA-overexpressing Mefs MAVS-Pex cells. The analysis of the Manders’ co-localization coefficient for these cells has shown that 8.60% of the vMIA co-localizes with the peroxisomal marker. Upon morphological and statistical analysis of the organelle’s morphology under these two conditions (in a similar way as previously shown for HepG2 and HFF cells), the results show that vMIA also induces peroxisomal fragmentation in Mefs MAVS-Pex cells (Fig. 5A,B). MAVS-dependent signalling events were stimulated in these cells by overexpressing a constitutively active version of RIG-I (GFP-RIG-I-CARD^27^,). Six hours after GFP-RIG-I-CARD transfection, the expression of two ISGs (IRF1 and viperin) was analysed by Western blot (Fig. 5C) and the production of their mRNA was quantified by RT-qPCR (Fig. 5D). Both analyses demonstrated a clear increase on the production of IRF1 and viperin upon GFP-RIG-I-CARD overexpression when compared with control un-stimulated Mefs MAVS-Pex cells. In the presence of vMIA, however, the production of IRF1 and viperin remained close to the levels observed in unstimulated cells. Collectively, these results indicate that vMIA disrupts MAVS signalling transduction from peroxisomes.

In order to demonstrate that these results were not due to a lower GFP-RIG-I-CARD expression in the presence of vMIA, we have analysed GFP-RIG-I-CARD expression levels in the presence and absence of the viral protein. Fig. S2A shows that, when vMIA is present, there is no decrease (there is even an increase) in the expression of GFP-RIG-I-CARD (6 h post-transfection) when compared to control cells where vMIA is absent. Similar results were obtained upon expression of a full version of RIG-I (GFP-RIG-I). We have also demonstrated that the presence of vMIA in the cells does not alter the production of GFP-RIG-I-CARD mRNA (Fig. S2B). In order to specifically detect the transfected (human) GFP-RIG-I-CARD mRNA and exclude the (mouse) endogenous RIG-I, this analysis was performed with primers against the human RIG-I.

**Figure 6:**
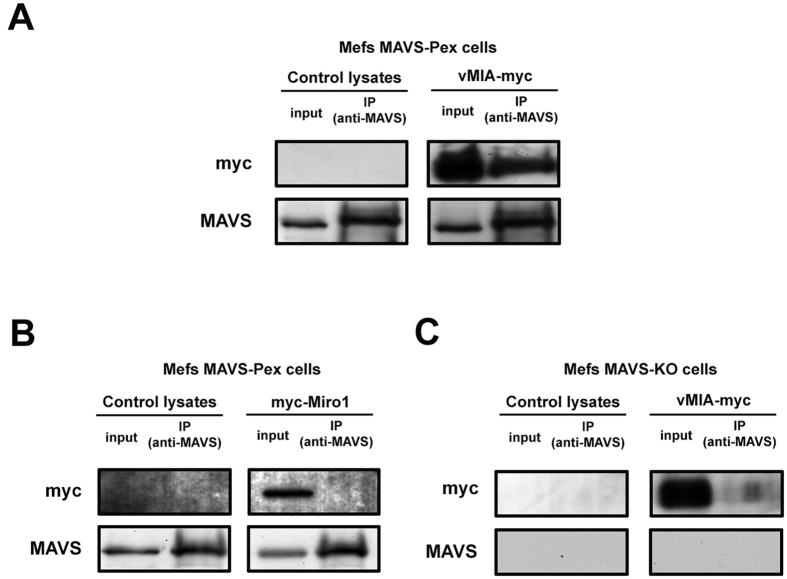
Interactions between peroxisomal MAVS and vMIA. (**A**) Co-immunoprecipitation analysis of the interaction between overexpressed vMIA-myc and endogenous MAVS in Mefs MAVS-Pex cells. Negative control was performed by immunoprecipitating non-transfected cells. The pull-down was performed using an antibody against MAVS. Western blot was performed with antibodies against MAVS and myc. Input represents total cell lysate and IP represents the immunoprecipitation. (**B**) As negative control, the mitochondrial myc-tagged Miro1 (myc-Miro1) was transfected in Mefs MAVS-Pex cells. The pull-down was performed using an antibody against MAVS. Western Blot was performed with antibodies against MAVS and myc. Input represents total cell lysate and IP represents the immunoprecipitation. (**C**) As negative control, vMIA-myc was transfected in Mefs MAVS-KO cells. The pull-down was performed using an antibody against MAVS. Western Blot was performed with antibodies against MAVS and myc. Input represents total cell lysate and IP represents the immunoprecipitation.

Up to now, no direct interaction between vMIA and MAVS has ever been demonstrated. In order to obtain more mechanistical details on the action of vMIA towards the peroxisomal-MAVS dependent signalling pathway, we analysed whether vMIA would be able to specifically associate with the peroxisomal MAVS. To that end, Mefs MAVS-Pex cells were transfected with vMIA-myc and, after 24 h, co-immunoprecipitation analyses were performed with an antibody against MAVS. As clearly shown in Fig. 6A, vMIA specifically interacts with the endogenous peroxisomal-MAVS (with an 18-fold increase when comparing the bands from the immunoprecipitation -IP- and the control). To demonstrate that MAVS is not interacting with the myc-tag of vMIA-myc, we have performed co-immunoprecipitation analyses in Mefs MAVS-PEX cells transfected with a myc-tagged protein that does not interact with MAVS (myc-Miro1) (Fig. 6B). Additionally, to demonstrate that vMIA-myc does not interact with the MAVS-antibody-coated beads, we performed co-immunoprecipitation analyses in Mefs MAVS-KO cells transfected with vMIA-myc (Fig. 6C)

### Peroxisomal fragmentation is not essential for the role of vMIA on the evasion of the immune response

**Figure 7:**
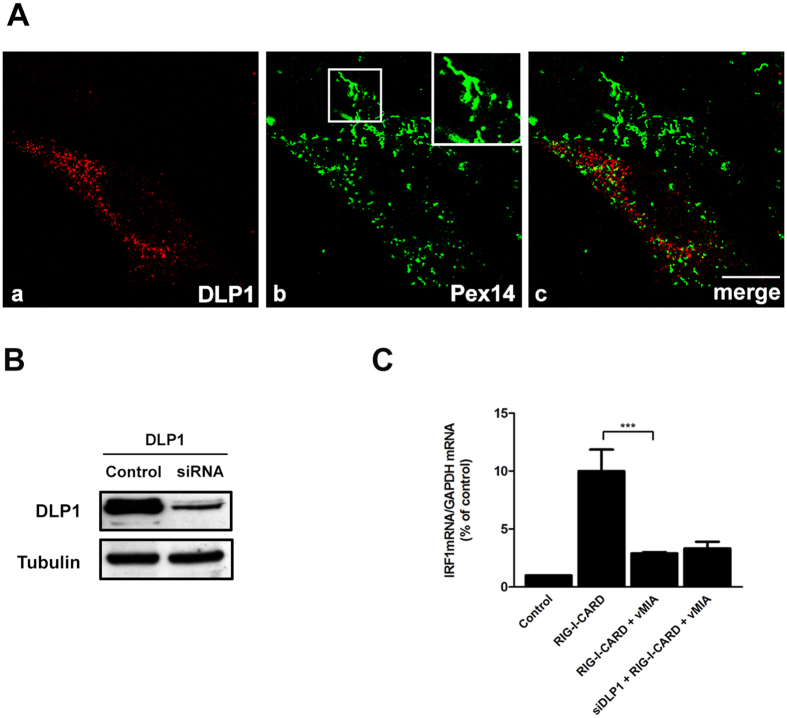
Peroxisomal fragmentation is not essential for vMIA’s inhibition of the peroxisomal-dependent antiviral signalling. (**A**) (a–c) Peroxisome morphology in DLP1-silenced Mefs MAVS-Pex cells (a) DLP1, (b) Pex14 and (c) merge image of a and b. Confocal images from immunofluorescence staining. Bar represents 10 μm. (**B**) Western blot analysis of the silencing of DLP1 in Mefs MAVS-Pex cells. Representative image of three independent experiments. (**C**) RT-qPCR analysis of the expression of IRF1 mRNA in Mefs MAVS-Pex cells stimulated with GFP-RIG-I-CARD in the presence of vMIA-myc and upon silencing of DLP1. Non-silenced cells, as well as cells not expressing vMIA-myc were used as controls. GAPDH was measured as control. Data represents the means ± SEM of three independent experiments. Error bars represent SEM. ***p < 0.001 in one-way ANOVA, with Bonferroni’s post test.

The localization of vMIA at peroxisomes and mitochondria has a strong effect on the organelles’ morphology, causing their fragmentation (Fig. 1, 5A and S1D^6^). Mitochondrial fragmentation was shown to be essential for the inhibition by vMIA of the mitochondrial MAVS-mediated signalling^9^. To determine if the ability of vMIA to induce peroxisome fragmentation similarly contributes to the disruption of peroxisomal-MAVS signalling, we sought to prevent vMIA-induced peroxisome fragmentation. To that end, we interfered with the peroxisome morphology by silencing DLP1, the cytoplasmic GTPase that mediates peroxisomal fission. We reasoned that if peroxisomal fragmentation is important for vMIA to block MAVS signalling, inhibiting DLP1 functions should permit the expression of ISGs, even in the presence of vMIA. To test this prediction, we silenced the expression of DLP1 in Mefs MAVS-Pex cells which were afterwards transfected with vMIA-myc (Fig. 7B). As shown in Fig. 7A, the peroxisomes from the silenced cells are highly elongated or even hypertubulated. These cells were then transfected with GFP-RIG-I-CARD and, after 6 h, the amounts of IRF1 mRNA were analysed by RT-qPCR. As shown in Fig. 7C, upon DLP1 silencing and in the presence of vMIA, the values of IRF1 mRNAs are statistically similar to the ones obtained upon GFP-RIG-I-CARD stimulation in the presence of vMIA. These results demonstrate that, when peroxisomal division is impaired, vMIA is still able to exert its inhibiting effect. Peroxisomal fragmentation is, hence, not essential for the inhibition of the peroxisomal MAVS-dependent antiviral signalling by HCMV.

## Discussion

Viruses have developed many sophisticated mechanisms to evade the cellular antiviral response. HCMV encodes vMIA, a powerful inhibitor of apoptosis that has also been shown to play a role on the inhibition of the mitochondrial MAVS-dependent antiviral signalling^28^,^29^. Up to now, this protein was described as localizing solely at mitochondria and all its reported metabolic functions were studied and justified based on its presence at this organelle^7^,^9^,^28^. Our results demonstrate for the first time that vMIA is also localized at peroxisomes, strongly affecting their morphology, and is able to interact with MAVS and to specifically inhibit the peroxisome-dependent antiviral signalling.

The localization of vMIA at peroxisomes was demonstrated by immunolocalization and confocal microscopy analyses, not only upon overexpression in hepatic cells (HepG2 cells), cells that are commonly infected by HCMV (HFF cells), DLP1-patient cells and Mefs MAVS-PEX cells, but also upon HCMV infection. Moreover, vMIA was also shown to be present in peroxisome-enriched fractions of HFF cells lysates and in the peroxisomal fractions of a density gradient of HepG2 cells.

Although the presence of viral proteins at peroxisomes is not unprecedented, most of the data available concerns the assembly of viral replication complexes of plant viruses (tombusvirus) at the peroxisomal membranes^30^. We, and others, have recently demonstrated that the Hepatitis C virus protein complex NS3-4A travels to peroxisomes and cleaves the peroxisomal MAVS, inhibiting the peroxisome-dependent immune response^21^,^31^,^32^. The HBx protein of *Hepatitis B virus* has also been shown to localize at peroxisomes and increase the invasiveness of hepatocellular carcinoma cells^33^. The N^pro^ from Pestivirus, that is able to bind and inactivate IRF3, was also found to partially localize at this organelle^34^.

The role of peroxisomes on the establishment of the cellular antiviral response has been demonstrated by Dixit *et al*.^15^, who have shown that peroxisomal and mitochondrial MAVS perform different but complementing functions within the antiviral response: while the peroxisomal MAVS induces a rapid and type I interferon-independent expression of defence factors providing short-term protection, the mitochondrial MAVS activates a type I interferon -dependent signalling pathway with delayed kinetics that amplifies and stabilizes the antiviral response. The same group has recently demonstrated that peroxisomes are the primary site of initiation of RLRs-induced type III interferon expression in a variety of human cell types^20^. One other recent report, however, somewhat contradicts these findings and show that, both peroxisomal and mitochondrial MAVS activation result in the production of type I and III interferons^31^.

Besides its peroxisomal localization, we have also demonstrated that vMIA interacts with Pex19, a cytoplasmic chaperone that is responsible, in concert with Pex3, for the transport of most peroxisomal membrane proteins to this organelle^25^,^35^,^36^,^37^. Our results suggest that HCMV highjacks the peroxisomal proteins’ targeting machinery to its own benefit, in order to transport vMIA to this organelle, upon exiting from the ER. Previous results with plant viruses (tombusvirus) have shown that Pex19 is also used to transport viral replication proteins to this organelle^38^.

One of our most interesting results is the fact that the presence of vMIA at peroxisomes causes the organelle’s fragmentation, a phenomenon that was not only observed upon the protein’s overexpression but also during viral infection. However, intriguingly, this fragmentation was shown not to be crucial for vMIA’s role on the inhibition of the peroxisomal-dependent antiviral signalling. vMIA had already been shown to induce mitochondrial fragmentation but this fragmentation was demonstrated to be essential for the inhibition of the mitochondrial-dependent signalling pathway^9^,^10^. Peroxisomes and mitochondria share the main components of their division machinery (e.g. the tail-anchored membrane adaptors Fis1 and Mff that recruit the large dynamin-related GTPase DLP1), which appears to be an evolutionary conserved strategy among organisms^39^,^40^,^41^,^42^,^43^,^44^,^45^,^46^,^47^. The main reasoning for vMIA-induced mitochondrial fragmentation has, up to now, been based on its role as an anti-apoptotic protein. vMIA has been shown to interfere with Bax to prevent mitochondrial outer-membrane permeabilization^6^,^7^, as well as to mediate the release of ER Ca^2+^ stores into the cytosol, inducing mitochondrial fission^8^. However, no correlation between peroxisomes and apoptosis has ever been established and anti- or pro-apoptotic proteins such as Bax have never been found at this organelle.

Based on the current knowledge and our results, vMIA-induced peroxisomal fragmentation seems to be a phenomenon that is independent from its role on the inhibition of the cellular antiviral signalling. This constitutes a first clue indicating that vMIA may act at peroxisomes and mitochondria via distinct mechanisms. A particular role for vMIA at peroxisomes may be justified by the need of the virus to specifically combat the rapid peroxisome-dependent production of antiviral compounds.

Since mitochondrial fusion is required to enhance the interaction between MAVS and the ER STING, it has been suggested that the vMIA-induced mitochondrial fragmentation may cause the reduction of this association, dampening signalling downstream from MAVS^9^,^14^. However, as peroxisomes do not fuse^48^ and, even when elongated, assume a similar cellular distribution, it seems unlikely that peroxisomal fragmentation would decrease the association with the ER, substantiating the fact that vMIA induced peroxisomal fragmentation is not the main mechanism responsible for the signalling inhibition in this organelle.

Interestingly, we have also shown that vMIA is able to specifically interact with the peroxisomal MAVS. Hence, our results support a model in which the mechanisms of vMIA-induced peroxisomal fragmentation and dampening of the peroxisomal-dependent antiviral signalling are different from the ones occurring in mitochondria. At peroxisomes, vMIA interferes with MAVS, likely impairing the downstream role of this protein in the signalling cascade, independently of the organelle’s morphology.

Our results not only substantiate the role of peroxisomes as a platform for viral evasion from the cellular antiviral response, but also present a novel mechanism by which HCMV is able to specifically evade the rapid and short-term peroxisomal MAVS-dependent antiviral signalling.

## Materials and Methods

### Antibodies and Plasmids

Rabbit antibody directed to Pex14 (a kind gift from Dr. Crane, Griffith University, Brisbane, Australia) and mouse antibodies directed to catalase (Abcam, Cambridge, UK) and myc epitope (9E10, Santa Cruz Biotechnology, Dallas, Texas, USA) were used for morphological studies. Rabbit serum anti-vMIA, used to detect the protein in HCMV infected cells, was a kind gift from Dr. Ed Mocarski from Stanford University (California, USA). Rabbit antibody directed to myc (71D10, Cell Signalling Technology, Beverly, Massachusetts, USA), mouse antibody directed to myc (Santa Cruz, Heidelberg, Germany), mouse antibody directed against COXIV (Abcam, Cambridge, UK), mouse antibody directed against TOM20 (BD Bioscience, San Jose, California, USA), mouse antibody directed against BiP/GRP (BD Bioscience, San Jose, California, USA), rabbit antibody directed against ACOX1 (a kind gift from Dr.T. Hashimoto Shinshu University School of Medicine, Nagano, Japan), mouse antibody directed to TIM23 (BD Bioscience, San Jose, California, USA), mouse antibody directed to PMP70 (Sigma-Aldrich, St. Louis, Missouri, USA), mouse antibody directed to MAVS (E-3, Santa Cruz Biotechnology, Dallas, Texas, USA), mouse antibody directed to DLP1 (BD Bioscience, San Jose, California, USA), mouse antibody directed to Pex19 (Sigma-Aldrich, St. Louis, Missouri, USA) and rabbit antibody directed to RIG-I (H-300, Santa Cruz Biotechnology, Dallas, Texas, USA), were used for immunobloting. The anti-viperin mouse MaP.VIP (a kind gift from Dr. Peter Cresswell from Yale University, Connecticut, USA) and rabbit IRF1 (Santa Cruz Biotechnology, Dallas, Texas, USA) antibodies were used for immunobloting to measure the production of this two ISGs. Anti-actin mouse antibody (provided by Dr. Jockusch, Braunschweig University, Germany) and anti-α-tubulin mouse antibody (Sigma-Aldrich, St. Louis, Missouri, USA) were used for immunoblotting as loading controls. Species-specific anti-IgG antibodies conjugated to HRP (BioRad, Hercules, California, USA), IRDye 800CW and IRDye 680RD secondary antibodies (LI-COR Biotechonology, Cambridge, UK) or to the fluorophores TRITC (Jackson Immunoresearch, West Grove, Pennsylvania, USA) and Alexa 488 (Invitrogen, Waltham, Massachusetts, USA) were used.

The construct encoding vMIA-myc was a gift from Dr. Goldmacher (ImmunoGen Inc., Cambridge, Massachusetts, USA). The truncated version of RIG-I protein, GFP-RIG-I-CARD, containing the CARD domain (1 to 284 aa) and the full-length version of RIG-I protein, GFP-RIG-I, were kindly provided by Dr. Weber (Philipps-University Marburg, Germany). The construct encoding Pex19-YFP was kindly provided by Dr. P.U. Mayerhofer (University of Munich, Germany).

### Cell Culture, transfections and RNA interference experiments

HepG2 (obtained from American Type Culture Collection, HB-8065), Human foreskin fibroblasts (HFF) (obtained from European Collection of Cell Cultures), Mefs MAVS-Pex cells (described in^15^) and DLP1-patient cell lines (kindly provided by H. Waterham, Academic Medical Center, Amsterdam, The Netherlands)^22^ (the parents consented for the use of these cells for scientific purposes) were cultured in Dulbecco’s modified Eagle’s medium supplemented with 100 U/mL penicillin, 100 mg/mL streptomycin and 10% fetal bovine serum (all from PAA Laboratories GmbH, Germany) and incubated at 37 °C in atmosphere containing 5% CO_2_. Cells were seeded on sterile glass coverslips and transfected 24 h after plating.

HepG2 and HFF cells were transfected with DNA constructs by incubation with TurboFect (Thermo Scientific, Waltham, Massachusetts, USA), by electroporation using the ECM 630 Electro Cell Manipulator (BTX Harvard Apparatus, Holliston, Massachusetts, USA) or using the Neon^®^ Transfection System (Invitrogen, Carlsbad, CA) (1700V, Width:20, 1 pulse), according to the manufacturer’s instructions. Mefs MAVS-Pex cells were transfected using Lipo3000 (Invitrogen, Waltham, Massachusetts, USA). The transfections were performed according to the manufacturer’s instructions. HepG2 cells were also transfected using polyethylenimine (PEI 25 kDa, Polysciences INC, Eppelheim, Germany), in detail, 1 μg plasmid DNA was mixed with 5 μL of a 1 mg/mL stock solution of PEI. The resulting DNA/PEI solution was diluted in serum free medium in a ratio of 1:8.5, incubated for 15 min at RT and subsequently used for transfection. 24 h after transfection, cells were trypsinized and collected by centrifugation at 500× g. DLP1-patient cells and also HFF were microporated with DNA using the Neon^®^ Transfection System (Invitrogen, Carlsbad, CA) (1700V, Width:20, 1 pulse), according to the manufacturer’s instructions. Cells were harvested and fixed from 6 to 24 h after transfection.

To knock-down the expression of DLP1 by RNA interference, a 21-nucleotide small interfering RNA (siRNA) duplex (pre-designed siRNA from Ambion - Waltham, Massachusetts, USA, according to^47^) was transfected in Mefs MAVS-Pex cells by incubation with Lipofectamine RNAiMax (Invitrogen, Waltham, Massachusetts, USA).

### Viral infection and virus stock preparation

HFF cells were cultured on sterile glass coverslips and infected with 5 p.f.u./cell HCMV laboratory strain AD169. After 8 h post-infection, cells were washed with PBS and fixed with 4% paraformaldehyde.

The HCMV laboratory strain AD169 was obtained from Dr. John Sinclair (University of Cambridge, United Kingdom). To prepare virus stocks of AD169 virus HFF cells were infected at a multiplicity of infection (MOI) of 0.01. After virus adsorption for one hour, infected cells were cultured at 37 °C and medium was collected every three days. Pre-cleared supernatants were centrifuged two hours at 12000 rpm at room temperature. Virus aliquots were stored at −80 °C. Virus stock titers were determined by plaque assay. Briefly, HFF cells were cultured with 10-fold dilutions of virus suspension and allowed to absorb for 1 h. Cells were then cultured with complete medium containing 10% carboxymethylcellulose (CMC) for 10–15 days. Cellular monolayers were fixed in 4% paraformaldehyde and stained with 0.1% toluidine blue. Quantification of the viral plaques was performed using a dissecting microscope.

### Immunofluorescence and microscopy analyses

Cells were processed for immunofluorescence as in^49^. In short, cells grown on glass coverslips were fixed with 4% paraformaldehyde in PBS, pH 7.4, for 20 min, permeabilized with 0.2% Triton X-100 for 10 min, blocked with 1% BSA solution for 10 min and incubated with the indicated primary and secondary antibodies for 1 h each. Finally cells were mounted in slides, using Mowiol 4-88 (AppliChem Inc. St. Louis, Missouri, USA) containing n-propylgallate (Sigma-Aldrich, St. Louis, Missouri, USA). Fixed samples were examined using an Olympus IX-81 inverted microscope (Olympus Optical Co. GmbH, Hamburg, Germany) equipped with the appropriate filter combinations and a 100x objective (Plan-Neofluar, 100×/1.35 oil objective). Confocal images were acquired using a Zeiss LSM 510 confocal microscope (Carl Zeiss, Oberkochen, Germany) using a Plan-Apochromat 63× and 100×/1.4 NA oil objectives, a 561 nm DPSS laser and the argon laser line 488 nm (BP 505–550 and 595–750 nm filters). All the confocal images presented in this manuscript represent a single plane, with the exception of the one where we present deconvolution analyses, Fig. 1C, were a z-stack was made. Images were processed using LSM 510 software (Carl Zeiss MicroImaging, Inc.). Digital images were optimized for contrast and brightness using Adobe Photoshop (Adobe Systems, San Jose, CA, USA). The Manders’ co-localization coefficient was applied to quantify the co-localization percentages between vMIA and the peroxisomes. After cropping the region of interest (ROI) from selected cells, channels were split, and quantifications were performed using the JACoP plugin (ImageJ, Bethesda, MD, USA) with a manually set threshold^50^.

Quantification analysis of the area of peroxisomes in HepG2 cells was performed using the Spot Detector plug-in^51^ from Icy Bioimage Analysis Software created by the Quantitative Image Analysis Unit at Institute Pasteur (Paris, France)^23^.

To generate high resolution images of the vMIA localization at the hypertubulated peroxisomes from DLP1-patient cells, deconvolution microscopy was performed. Fixed cells were examined by confocal microscopy. Using the 488 and 543 nm laser lines, z-stacks of transfected cells were generated (8× zoom) using the optimal number of slices suggested by the program. Oversaturation of signals was avoided by adjusting of respective photomultipliers. Image deconvolution and 3D rendering was performed using Huygens Professional Software (Scientific Volume Imaging, Hilversum, The Netherlands).

### Cell fractionations

For the cellular fractionation, HFF cells expressing vMIA-myc were homogenized in homogenization buffer (5 mM MOPS, pH 7.4, 250 mM sucrose, 1 mM EDTA, protease inhibitor mixture) by passing gently through a 26.5-gauge syringe needle. The homogenate was cleared by centrifugation (500× g for 5 min). Heavy mitochondria were subfractionated by centrifugation at 2500× g for 10 min (Mito). The organelle pellet was then gently resuspended in homogenization buffer and the supernatant was centrifuged again at 25,000× g to obtain the peroxisome-enriched fraction (PO). Pellet with the enriched PO fraction was gently resuspended in homogenization buffer and the supernatant was collected (Cyto). The organelle fractions were analysed by SDS-PAGE and immunoblotting.

For the density gradient, HepG2 cells expressing vMIA-myc were suspended in homogenization buffer (250 mM sucrose, 5 mM MOPS, 1 mM EDTA, 2 mM PMSF, 1 mM DTT, 1 mM ɛ-aminocaproic acid, pH 7.4) and homogenized by shearing through a syringe with a 27-gauge needle. Thereafter, cellular debris and nuclei were separated from the post-nuclear supernatant (PNS) by centrifugation at 600× g, 10 min, 4 °C. The PNS was subsequently centrifuged at 2000× g yielding the pellet of heavy mitochondria. The corresponding supernatant was subjected to another centrifugation at 20000× g to produce the peroxisome-enriched light mitochondrial pellet. This pellet was resupended in homogenization buffer and placed onto a linear Nykodenz gradient with a density between 1.14–1.19 g/mL. The gradients were centrifuged at a velocity of 100000× g for 3 h and collected in 12 equal sized fractions. For further analysis the individual fractions were pelleted by centrifugation and suspended in an appropriate volume of homogenization buffer. Protein concentrations were determined by the Bradford method; only fractions containing significant amounts of protein were subjected to immunoblotting.

### Immunoprecipitation analyses

To study the interaction of Pex19 and vMIA, HepG2 cells were co-transfected with Pex19-YFP and vMIA-myc by electroporation, using the ECM 630 Electro Cell Manipulator. For immunoprecipitation of Pex19-YFP the GFP-Trap_M kit (Chromotek, Planegg-Martinsried, Germany), consisting of a high quality GFP-binding protein coupled to a monovalent matrix of magnetic agarose beads, was used. Co-transfection of vMIA-myc and GFP-N1 was used as negative control. Twenty four hours post-transfection, the cell pellets were incubated with lysis buffer (10 mM Tris-HCl, pH 7.5, 150 mM NaCl, 0.5 mM EDTA, 0.5% NP-40 and a protease-inhibitor mix). The lysate was cleared by centrifugation (17,000× g, 15 min) and diluted with dilution buffer (10 mM Tris-HCl, pH 7.5, 150 mM NaCl, 0.5 mM EDTA and a protease-inhibitor mix). Protein concentrations were determined by the Bradford assay (BioRad, Hercules, CA, USA). Ice-cold dilution buffer was used to equilibrate beads and then cell lysate was incubated for 2 h at 4 °C on a rotary mixer. Beads were washed 3 times with dilution buffer and then resuspended in 3× SDS-sample buffer and boiled for 10 min to elute bound proteins.

With the same purpose, HFF cells were transfected with vMIA-myc or with vMIA-myc and Pex19-YFP, using the Neon^®^ Transfection System (Invitrogen, Carlsbad, CA) (1700V, Width:20, 1 pulse), according to the manufacturer’s instructions. For immunoprecipitation of vMIA-myc the Protein G Magnetic beads kit (Millipore, Massachusetts, USA) was used. HFF cell lysate was used as negative control. After 24 h of transfection cell pellets were incubated with lysis buffer (10 mM Tris-HCl, pH 7.5, 150 mM NaCl, 0.5 mM EDTA, 0.5% NP-40 and a protease-inhibitor mix). Protein concentrations were determined by Bradford assay. The cell lysate was incubated with myc antibody for 2 h at 4 °C on a rotary mixer and then the beads were added to the mixture and rotated for 10 min at room temperature. The complex was washed 3 times with PBS containing 0.1% Tween 20 and then resuspended in 3x SDS-sample buffer and boiled for 10 min to elute bound proteins. For immunoprecipitation of vMIA-myc and Pex19-YFP the Dynabeads Protein G beads (Invitrogen, Waltham, Massachusetts, USA) were used. HFF cells transfected with vMIA-myc were used as negative control. Cells lysates were prepared as described above, as well as protein concentration. Then, cells lysates incubated with GFP antibody overnight at 4 °C on a rotary mixer. Beads were added to the mixture and rotated for 2 h at 4 °C on a rotary mixer. The complex was washed 3 times with PBS containing 0.1% Tween 20 and then resuspended in 3x SDS-sample buffer and boiled for 10 min to elute bound proteins. All the immunoprecipitated samples were separated by running in a 12,5% SDS-polyacrylamide gel.

To study the interaction of MAVS and vMIA, Mefs MAVS-Pex cells were transfected with vMIA by Lipo3000. To provide negative controls, Mefs MAVS-Pex cells were transfected with myc-Miro1 and Mefs MAVS-KO cells were transfected with vMIA-myc. For immunoprecipitation of vMIA-myc and myc-Miro1 the Dynabeads Protein G Magnetic beads kit was used. Untransfeted Mefs MAVS-Pex cells or Mefs MAVS-KO cells were also used as negative control for each immunoprecipitation. The procedure was performed as indicated above, using an antibody against MAVS for the pull-down step.

### Gel Electrophoresis and Immunoblotting

Cells were lysed with specific lysis buffer (25 mM Tris-HCl, pH 8.0, 50 mM sodium chloride, 0.5% sodium deoxycholate, 0.5% Triton X-100 and a protease-inhibitor mix). To improve protein extraction, samples were passed 20 times through a 26-gauge syringe needle and then incubated on a rotary mixer for 30 min at 4 °C. After clearing by centrifugation (17000× g, 15 min), protein concentrations were determined using the Bradford assay. Protein samples were separated by SDS-PAGE on 10% or 12.5% polyacrylamide gels, transferred to nitrocellulose (PROTAN^®^, Whatman^®^, Dassel, Germany) using a wet or a semidry system (BioRad, Hercules, California, USA), and analyzed by immunoblotting. Immunoblots were processed using specific primary antibodies, and either HRP-conjugated secondary antibodies and enhanced chemiluminescence reagents (GE Healthcare, Waukesha, Wisconsin, USA) or IRDye 800CW and IRDye 680RD secondary antibodies (LI-COR Biotechonology, Cambridge, UK). For quantification, immunoblots were scanned with a Bio-Rad GS-800 (BioRad, Hercules, California, USA) calibrated imaging densitometer or with Odyssey CLx (LI-COR Biotechonology, Cambridge, UK) and processed using the volume tools from Bio-Rad Laboratories Quantity One software (BioRad, Hercules, California, USA). The background intensity was calculated using the local background subtraction method.

### RNA extraction, cDNA synthesis and quantitative real-time polymerase chain reaction

Total RNA was isolated from Mefs MAVS-Pex cells using TriFast reagent (Peqlab, VWR International GmbH, Erlangen, Germany). RNA concentration was determined using NanoDrop 1000 (Thermo Scientific, Waltham, Massachusetts, USA). 1–3 μg of total RNA and M-MuLV reverse transcriptase (New England Biolabs, Ipswich, Massachusetts, USA) was used to perform cDNA synthesis. Real-time polymerase chain reaction was performed with duplicates using iTaq™ Universal SYBR^®^ Green Supermix (BioRad, Hercules, California, USA) and reactions were run on Applied Biosystems^®^ 7500 Real-Time PCR System (Applied Biosystems, Waltham, Massachusetts, USA). Primer sequences were designed using Beacon Designer™ 7 (Premier Biosoft, Palo Alto, California, USA) for the IRF1, Viperin, Pex14, and GAPDH mouse genes, as well as for the RIG-I human gene. The oligonucleotides used for IRF1 were 5′-GGTCAGGACTTGGATATGGAA-3′ and 5′-AGTGGTGCTATCTGGTATAATGT-3′; for viperin the 5′-TGTGAGCATAGTGAGCAATGG-3′ and 5′-TGTCGCAGGAGATAGCAAGA-3′; for Pex14 the 5′-GCCACCACATCAACCAACT-3′ and 5′-GGGAAGGAGGGAACTGTC-3′; for mouse GAPDH the 5′-AGTATGTCGTGGAGTCTA-3′ and 5′-CAATCTTGAGTGAGTTGTC-3′; and for human RIG-I the 5′-CTGGACCCTACCTACATC-3′ and 5′- CCAACAGGAACTTGAGAA-3′. GAPDH was used as a reference gene. For gene expression analysis, 2 μL of 1:10 diluted cDNA was added to 10 μL of 2× iTaq SYBR Green Master Mix (BioRad, Hercules, California, USA) and the final concentration of each primer was 250 nM in 20 μL total volume. The thermocycling reaction was initiated by activation of iTaq DNA Polymerase by heating at 95 °C during 3 min, followed by 40 cycles of a 12 s denaturation step at 95 °C and a 30 s annealing/elongation step at 60 °C. The fluorescence was measured after the extension step using the Applied Biosystems software (Applied Biosystems, Waltham, Massachusetts, USA). After the thermocycling reaction, the melting step was performed with slow heating, starting at 60 °C and with a rate of 1%, up to 95 °C, with continuous measurement of fluorescence. Data analysis was performed using the 2^−ΔΔCT^ method.

### Statistical analyses

Statistical analysis was performed in Graph Pad Prism 5 (GraphPad Software, Inc., La Jolla, California, USA). Quantitative data are presented as mean ± standard error mean (SEM). Differences among groups were analyzed by one-way ANOVA, followed by Bonferroni’s multiple comparison test; comparisons between two groups were made by Student’s *t* test. P values of ≤0.05 were considered as significant.

## Additional Information

**How to cite this article**: Magalhães, A. C. *et al*. Peroxisomes are platforms for cytomegalovirus’ evasion from the cellular immune response. *Sci. Rep.* **6**, 26028; doi: 10.1038/srep26028 (2016).

## Supplementary Material

Supplementary Information
