# Supplementary material for: Peroxisomes are platforms for cytomegalovirus’ evasion from the cellular immune response
Source: Sci Rep. 2016 May 16;6:26028. doi: 10.1038/srep26028 (PMC4867596; doi:10.1038/srep26028)
Supplement: Supplementary Information [file srep26028-s1.pdf]

Peroxisomes are platforms for cytomegalovirus' evasion from the cellular immune response

Ana Cristina Magalhães[1#], Ana Rita Ferreira[1#],Sílvia Gomes[1], Marta Vieira[1], Ana Gouveia[1], Isabel Valença[1], Markus Islinger[2], Rute Nascimento[3], Michael Schrader[4], Jonathan C. Kagan[5] and Daniela Ribeiro[1*]

co-authorship

Affiliations: [1]Institute for Research in Biomedicine – iBiMED, Department of Medical Sciences & Department of Biology, University of Aveiro, Aveiro, Portugal, [2]Neuroanatomy, Center for Biomedicine and Medical Technology Mannheim, University of Heidelberg, Heidelberg, Germany, [3]Infections and Immunity Laboratory, Instituto Gulbenkian de Ciência, Oeiras, Portugal, [4]College of Life and Environmental Sciences, Biosciences, University of Exeter, Exeter, Devon, UK, [5]Division of Gastroenterology, Boston Children's Hospital and Harvard Medical School, Boston, MA, USA

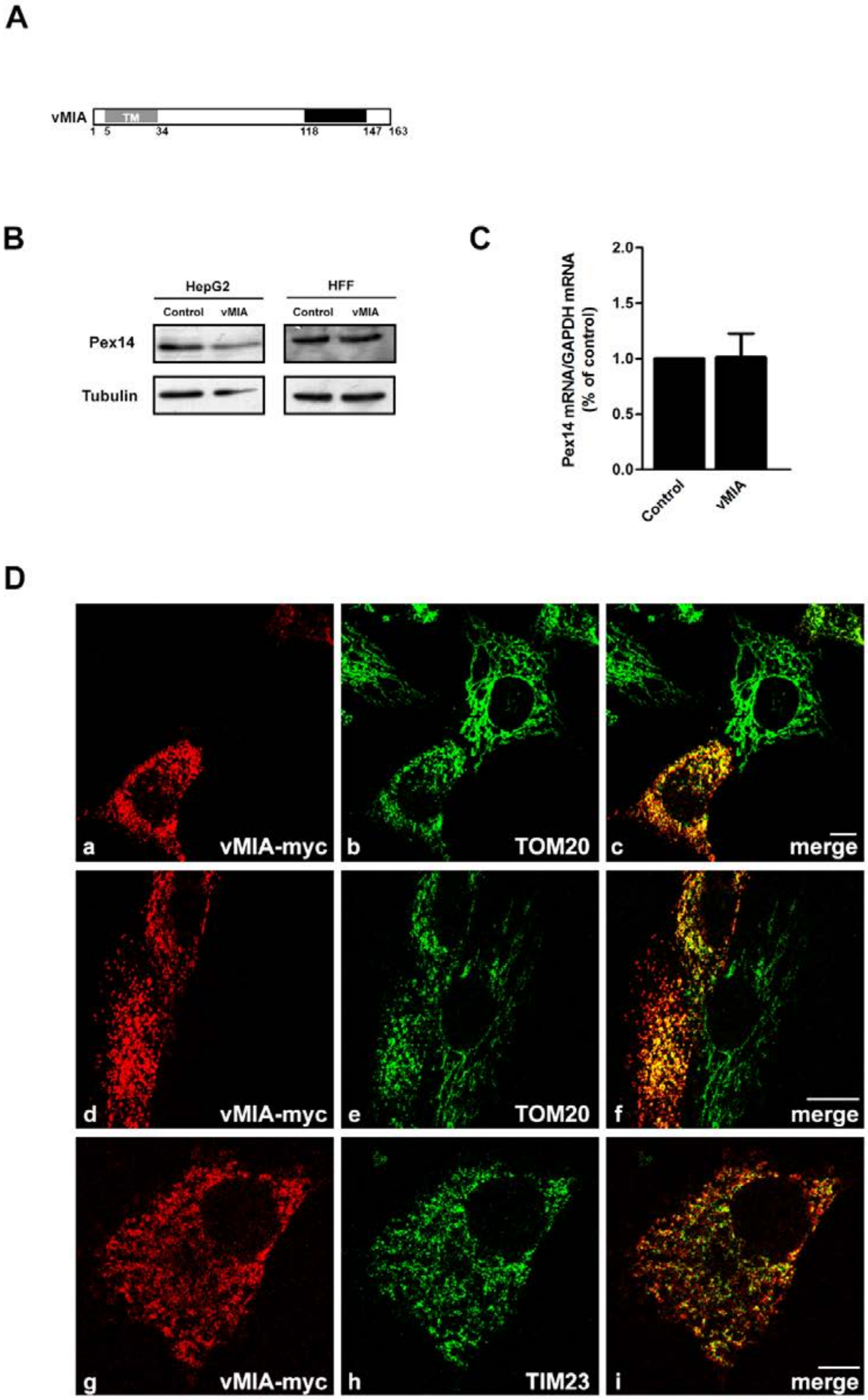

Figure S1– A- Schematic representation of vMIA topology. vMIA is constituted by 163 amino acids. In the N-terminal it contains a transmembrane domain (TM), localized between the amino acids 5 – 34. The C-terminal functional domain (in black) is located between the amino acids 118-147. Adapted from

Goldmacher V. 2002. B– Western blot analysis of the Pex14 expression in the absence or presence of vMIA in HepG2 and HFF cells. Representative image of three independent experiments. Tubulin was used as a loading control. C- RT-qPCR analysis of Pex14 mRNA production in the absence or presence of vMIA in Mefs MAVS-Pex cells. GAPDH was used as control. Data represents the means ± SEM of three independent experiments. Error bars represent SEM. D- (a-c) vMIA intracellular localization in HepG2 cells (a) vMIA-myc, (b) TOM20 and (c) merge image of a and b. (d-f) vMIA intracellular localization in HFF cells (d) vMIA-myc, (e) TOM20 and (f) merge image of d and e. (g-i) vMIA intracellular localization in Mefs MAVS-Pex cells (g) vMIA-myc, (h) TIM23 and (i) merge image of g and h. Confocal images from immunofluorescence staining. Bars represent 10 µm.

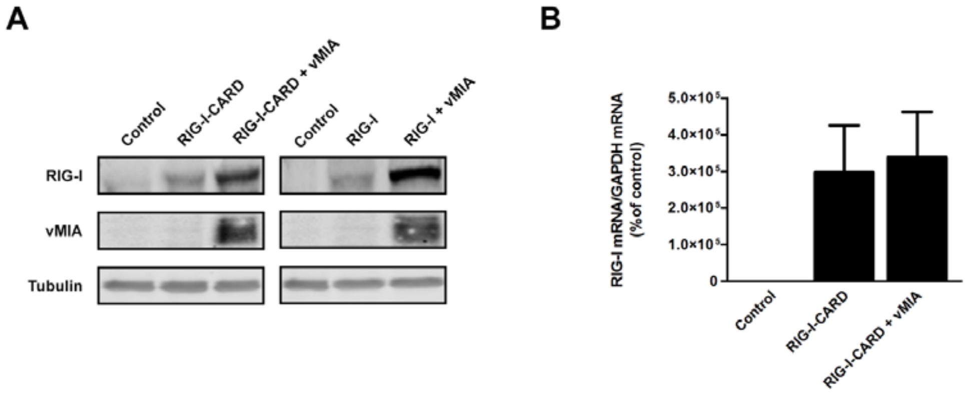

Figure S2 – A- Western blot analysis of the production of GFP-RIG-I-CARD and GFP-RIG-I in Mefs MAVS-Pex cells in the presence or absence of vMIA. Tubulin was used as a loading control. B- RT-qPCR analysis of RIG-I mRNA in Mefs MAVS-Pex cells stimulated with GFP-RIG-I-CARD in the presence or absence of vMIA (performed with primers annealing with the human RIG-I, in order to solely analyse the transfected human GFP-RIG-I-CARD). GAPDH was used as control. Data represents the means ± SEM of three independent experiments. Error bars represent SEM.
